# Is local radiotherapy a viable option for patients with an opening of the ventricles during surgical resection of brain metastases?

**DOI:** 10.1186/s13014-020-01725-x

**Published:** 2020-12-10

**Authors:** Sophia Scharl, Kerstin A. Kessel, Christian Diehl, Jens Gempt, Bernhard Meyer, Claus Zimmer, Christoph Straube, Stephanie E. Combs

**Affiliations:** 1 Department of Radiation Oncology, Technische Universität München (TUM), Ismaninger Straße 22, Munich, Germany; 2 Institute of Radiation Medicine (IRM), Helmholtz Zentrum München, Ingolstädter Landstraße 1, Neuherberg, Germany; 3 Deutsches Konsortium Für Translationale Krebsforschung (DKTK), Partner Site Munich, Munich, Germany; 4 Department of Neurosurgery, Technische Universität München (TUM), Ismaninger Straße 22, Munich, Germany; 5 Department of Diagnostic and Interventional Neuroradiology, Technische Universität München (TUM), Ismaninger Straße 22, Munich, Germany

**Keywords:** Local radiotherapy, Brain metastases, Ventricular opening, Hypofractionated stereotactic radiotherapy, Leptomeningeal disease

## Abstract

**Background:**

Local hypofractionated stereotactic radiotherapy (HFSRT) of the resection cavity is emerging as the standard of care in the treatment of patients with a limited number of brain metastases as it warrants less neurological impairment compared to whole brain radiotherapy. In periventricular metastases surgical resection can lead to an opening of the ventricles and subsequently carries a potential risk of cerebrospinal tumour cell dissemination. The aim of this study was to assess whether local radiotherapy of the resection cavity is viable in these cases.

**Methods:**

From our institutional database we analyzed the data of 125 consecutive patients with resected brain metastases treated in our institution with HFSRT between 2009 and 2017. The incidence of LMD, overall survival (OS), local recurrence (LC) and distant recurrence were evaluated depending on ventricular opening (VO) during surgery.

**Results:**

From all 125 patients, the ventricles were opened during surgery in 14 cases (11.2%). None of the patients with VO and 7 patients without VO during surgery developed LMD (*p* = 0.371). OS (*p* = 0.817), LC (*p* = 0.524) and distant recurrence (*p* = 0.488) did not differ in relation to VO during surgical resection. However, the incidence of distant intraventricular recurrence was slightly increased in patients with VO (14.3% vs. 2.7%, *p* < 0.01).

**Conclusion:**

VO during neurosurgical resection did not affect the outcome after HFSRT of the resection cavity in patients with brain metastases. Particularly, the incidence of LMD was not increased in patients receiving local HFSRT after VO. HFSRT can therefore be offered independently of VO as a local treatment of tumor bed after resection of brain metastases.

## Introduction

Local therapies, such as surgery and radiosurgery, have become the standard of care in patients with a limited number of brain metastases as they combine excellent outcomes with a reduction of neurological side effects compared to whole brain radiotherapy (WBRT) [1–3]. Surgery of solitary brain metastases has been shown not only to reduce seizures, symptoms of intracranial pressure and neurological deficits but also to improve overall survival (OS) in patients with good performance status and controlled systemic disease compared to WBRT [4].

In order to avoid cognitive decline, adjuvant radiotherapy to the resection cavity is also shifting towards a local approach [2, 3, 5–7]. Local radiotherapy to the resection bed can be administered by radiosurgery with single doses of 15–22 Gy or hypofractionated stereotactic radiotherapy (HFSRT) [2, 3, 6, 8–11]. Previously, our group has demonstrated excellent local control after HFSRT to the resection cavity of approximately 80% [6, 8]. Furthermore, we showed that distant intracranial recurrences and even most local failures can be salvaged successfully without an excessive risk for radionecrosis [12].

The reduction of neurological side effects of local radiotherapy comes at a price. Both, surgery and local radiotherapy entail higher rates of leptomeningeal disease (LMD) and distant failure compared to WBRT [13]. In the case of surgery without adjuvant radiotherapy local control is reduced as well [14]. The risk of LMD development after surgical resection of brain metastases occurred particularly in patients with posterior fossa metastases and could be reduced significantly by changing the resection technique to “en-block” resection as compared to “piecemeal” [4, 15–17]. Different factors may contribute to an increased LMD manifestation including the tumors natural biology and local radiotherapy leading to higher incidences of distant tumor progression resulting in LMD.

The contamination of cerebrospinal fluid during the course of surgery, nevertheless, should not be ignored as a potential trigger mechanism leading to an increase in LMD after surgical resection [11, 15, 16]. In periventricular metastases surgical resection can lead to an opening of the ventricles. Subsequently it carries a potential risk of cerebrospinal tumor cell dissemination [17–21]. The aim of this study was to assess whether local HFSRT of the resection cavity is a viable option in patients with resected brain metastases in which VO was necessary during surgery.

## Methods

All patients were treated in accordance with the Declaration of Helsinki. A written informed consent in the use of scientific data was obtained by all patients. This study was approved by the Ethics Committee of the Technical University of Munich.

### Radiotherapy

The clinical target volume (CTV) was defined as the resection cavity (encompassing residual tumor, if present) plus a safety margin of 2–3 mm. Planning target volume (PTV) was generated with an additional margin of 1–2 mm to the CTV. 35 Gy (or 30 Gy, if adjacent to brain stem) were applied in daily doses of 5 Gy. Radiotherapy was applied on 5 days per week. Dose prescription was to the 95–100% isodose line. The prescription dose corresponds to the standard scheme for the local irradiation in our institute. Its effectiveness and safety has been published previously [8, 22]. The biologically effective dose (BED) that is equivalent to 35 Gy in daily doses of 5 Gy depends on the alpha/beta ratio of the irradiated tissue. For breast cancer, lung cancer, and GI cancer cells, with estimated alpha/beta ratios of 4–8, this corresponds to a BED of 65.6 to 96.3 Gy. When assuming an alpha/beta of 2 for healthy brain tissue, the equivalent BED is 122.5 Gy [25]. Further metastases were treated with simultaneous or sequential stereotactic radiosurgery with a dose of 20 Gy prescribed to the 80%-Isodose line or hypofractionated RT with 35 (or 30 Gy, if adjacent to brain stem) in daily doses of 5 Gy. The majority of treatment planning was performed using 6–9 coplanar and non-coplanar beams by iPlan treatment planning software (BrainLAB AG, Munich, Germany). If lesions were close to critical organs at risk, IMRT planning was carried out by Eclipse software (version 13; Varian Medical Systems, Palo Alto, CA, USA).

Irradiation was performed with a Clinac Trilogy linear accelerator equipped with a 120 HD multi-leaf collimator (Varian Medical Systems, Palo Alto, CA, USA) and 6 MV photons. A high precision treatment set-up was applied using a frameless thermoplastic mask system (BrainLAB AG, Munich, Germany). Daily image-guided radiotherapy was performed with the ExacTrac stereoscopic X-ray imaging system.

### Outcome and definitions

Local recurrence was defined as a recurrence at the site of the initial metastases, distant recurrence as a recurrence elsewhere in the brain. Recurrence was documented if stated as such in the MRI report. LMD was documented if stated as such in the MRI report or in cases with detection of tumor cell in cerebrospinal fluid (CSF). Local meningeal enhancement was defined as enhancement in no more than one location and of a length of less than 3 cm along the meninges. Local meningeal enhancement was not considered LMD without the presence of positive CSF. VO was documented if stated as such in the surgical report. Proximity to the ventricles was defined as ≤ 3 mm between the edge of metastasis and the closest point of any of the four ventricles. Distant ventricular metastasis was defined as a solitary local intraventricular metastases without LMD.

### Statistical evaluation

Time to local recurrence, distant cerebral recurrence, LMD and distant intraventricular metastases were calculated from the starting day of radiotherapy until the date of tumor recurrence, LMD occurrence, or detection of distant intraventricular metastases, respectively. In patients with more than one resection cavity, each cavity was regarded individually in the calculation of local recurrence. For the evaluation of overall survival (OS), the time interval between the starting day of radiotherapy to the date of death or the last contact was calculated.

Continuous data were expressed as means ± standard deviation or median and range, categorical data as frequency counts or percentages. Categorical data were compared by chi-square test. OS and recurrence rates were calculated by Kaplan–Meier-method. For comparison of survival distributions, the log-rank test was used. A p-value of 0.05 was defined as the threshold for statistical significance within a confidence interval of 95%. All calculations and figures were done with the software packages SPSS 23 (IBM, USA).

## Results

### Patients

125 patients with 130 resection cavities treated with HFSRT after resection of 1–3 brain metastases between 2009 and 2017 in our institution, in which information on ventricular opening was available, were included in this study. Median age was 63 years (range 19–85 years), most common primaries were non-small cell lung cancer (30 cases/24.0%); gastrointestinal cancers (23 cases/18.4%) and breast cancer (23 cases/18.4%). 28 of the 125 patients had 1–2 further metastases that did not require surgery due to their small volume and were treated by radiotherapy only. They were irradiated sequentially by stereotactic radiosurgery or hypofractionated RT as described above.

The only significant difference between the groups was the preoperative diameter of the metastases (Table1).

**Table 1:** Patients’ characteristics

	Ventricular opening (VO)		
	VO	No VO	*p*-value
Mean age	59.9 ± 14.6	61.3 ± 13.5	0.710
*Histologies*			
Breast cancer	2 (14%)	21 (19%)	
NSCLC	3 (21%)	27 (24%)	
GI tract	2 (14%)	21 (19%)	
Melanoma	2 (14%)	17 (15%)	
other	5 (35%)	25 (23%)	0.871
*Location*			
Supratentorial	10 (71%)	82 (74%)	
Infratentorial	4 (29%)	25 (23%)	
Both	0	4 (4%)	0.701
*RPA class*			
1	3 (21%)	23 (21%)	
2	7 (50%)	68 (61%)	
3	4 (29%)	13 (12%)	
Unknown	0	7 (6%)	0.263
*GPA*			
< 2	1(1%)	24 (22%)	
2–3	8 (57%)	46 (41%)	
> 3	1 (1%)	14(12%)	
Unknown	4(29%)	27 (24%)	0.617
*Number of metastases*			
1	9 (64%)	84 (76%)	
2	5 (36%)	20 (18%)	
3	0	7 (6%)	0.218
*Year of treatment*			
2009–2011	3 (21%)	25 (23%)	
2012–2014	4 (29%)	28 (25%)	
2015–2017	7 (50%)	55 (5%)	0.873
*Karnosfky performance score*			
100–90	3 (21%)	52 (47%)	
80–70	6 (43%)	41 (37%)	
≤ 60	4 (29%)	13 (12%)	
Unknown	1 (1%)	5 (5%)	0.122
*Residual tumor*			
Yes	7 (50%)	68 (61%)	
No	5 (36%)	24 (22%)	
Unknown	2 (14%)	19 (17%)	0.512
Ø metastasis (mm)	36.3 ± 7.7	29.5 ± 11.5	0.047*

*Significant differences between the groups

### Incidence of ventricular opening

In 21.6% of patients (n = 27 of 125) the initial metastasis was located in proximity to the ventricles. The risk for VO was significantly increased in those metastases (*p* < 0.01): 11.2% of patients (n = 14 of 125) in the overall cohort and 44.4% of patients (n = 12 of 27) with metastases in proximity to the ventricular system, respectively, experienced VO during surgery.

### Overall survival

**Fig. 1:**
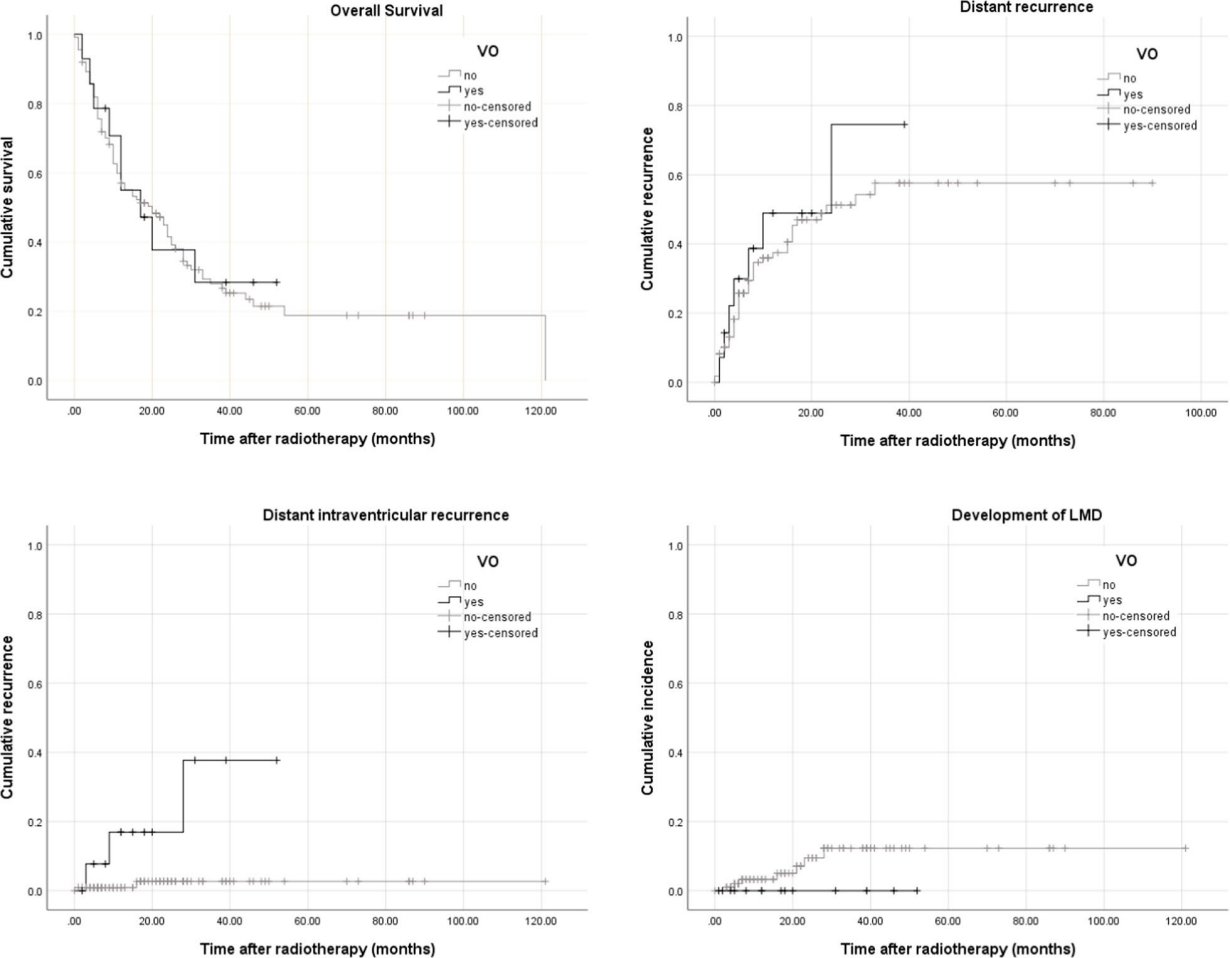
Kaplan–Meier Curves for overall survival, local recurrence, distant intraventricular recurrence and LMD

1- and 2-year OS in the complete cohort was 56.8% and 41.1%, respectively. Mean follow-up time was 49.6 months (± 4.5 months). There was no difference in OS depending on an opening of the ventricles during surgery (*p* = 0.817): 1- and 2-year OS was 55.0% and 37.7% for patients with VO compared to 57.0% and 41.4% for patients without VO (Fig. 1). A location of the initial metastases in the proximity to the ventricles was not significantly correlated with OS (*p* = 0.445).

### Local control

The 1- and 2-year local control rate in the complete cohort was 86.0% and 68.2%, respectively. There was no significant difference in local control between patients with and without VO (*p* = 0.524): Patients with VO had 1- and 2-year local control rates of 90.0% and 60.0% compared to 85.7% and 69.0% for patients without VO (Fig. 1). A location of the initial metastases in the proximity to the ventricles was not significantly correlated with local control (*p* = 0.167).

### Distant cerebral control

Overall 1- and 2-year distant control was 61.3% and 46.8%. There was no difference in distant control for patients with VO compared to those without (*p* = 0.488): 1- and 2-year distant control was 62.6% vs. 48.8% and 51.1% vs. 25.6% for patients without and with VO, respectively. Again, distant cerebral control was not significantly correlated with a location of the initial metastases in the proximity to the ventricles (*p* = 0.191).

### Leptomeningeal disease

LMD occurred in 7 of 125 patients (5.6%). In 2 cases LMD was diagnosed by CSF cytology, in 5 cases clinical and radiological signs lead to the diagnosis. All of the cases of LMD occurred in patients without VO (*p* = 0.371) (Fig. 1). Mean time between diagnosis of LMD and death was 4.9 months (± 1.7 months) compared to 17.2 months (± 3.1 months) between diagnosis of any distant recurrence and death (*p* < 0.01). Time from surgery to the development of LMD was significantly correlated with OS in patients that developed LMD (*p* = 0.039).

### Risk factors for LMD

LMD occurred more frequently in patients with gastrointestinal tumors (15.0%) than in patients with NSCLC (0%) and patients with tumors classified as others (0%) (*p* = 0.035 and *p* = 0.045, respectively). The remaining cases of LMD occurred in patients with breast cancer (9.5%) and melanoma (11.8%). Patients with infratentorial metastases had a slightly higher LMD rate (11.5%) than patients with supratentorial metastasis (5.0%), however, without statistical significance (*p* = 0.418). The preoperative metastatic diameter was not correlated with the development of LMD (*p* = 0.985). A location of the initial metastases in the proximity to the ventricles was not significantly correlated with the development of LMD (*p* = 0.317).

### Distant intraventricular metastases

**Fig. 2:**
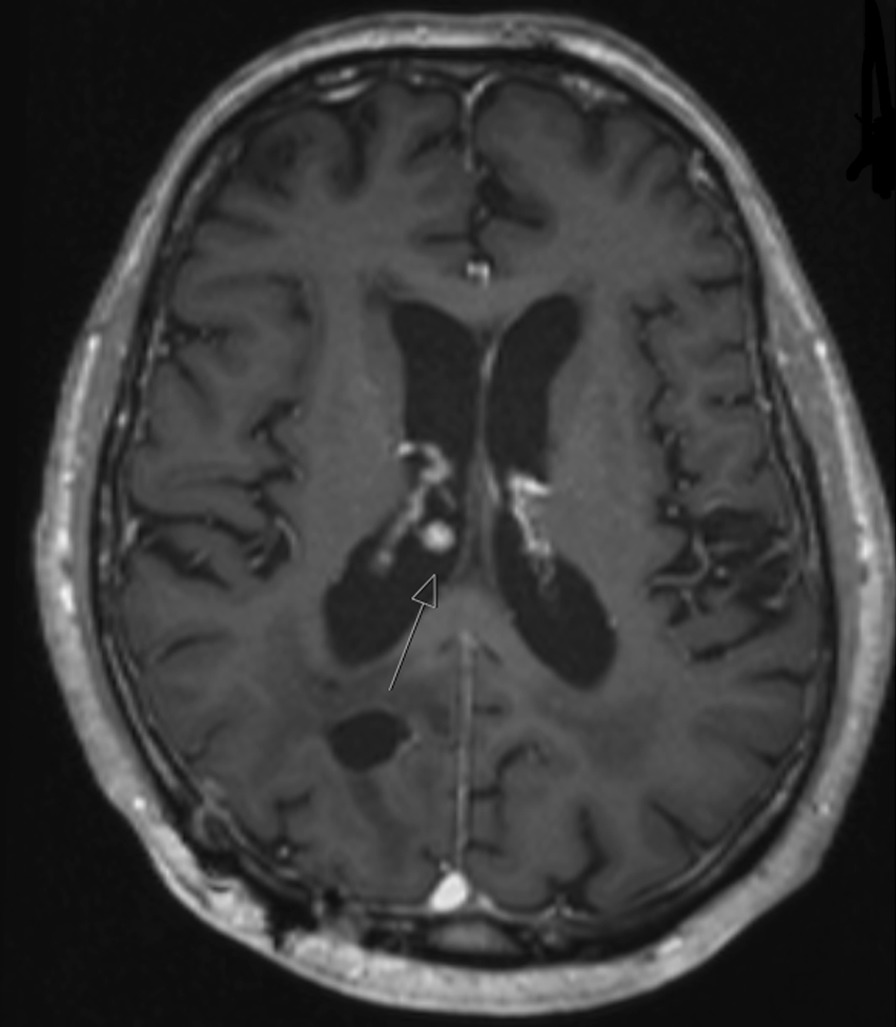
Distant intraventricular recurrence in a patient with VO during initial surgery

5 patients in the complete cohort developed distant intraventricular metastases. 14.3% (2 patients) of patients with VO and 2.7% (3 patients) without developed distant intraventricular metastases (*p* < 0.01) (Figs. 1 and 2). There was no significant difference in OS between patients with and without intraventricular recurrence (Median OS 29.6 months ± 8.5 months vs. 37.1 months ± 4.5 months, *p* = 0.690). A location of the initial metastases in the proximity to the ventricles was significantly correlated with distant intraventricular recurrence (*p* < 0.01).

### Radionecrosis

11.2% of patients (n = 14 of 125) developed radionecrosis. The risk for radionecrosis was not significantly associated with greater safety margins in this cohort (*p* = 0.657).

### Salvage treatment

At first recurrence, 7.8% of patients (n = 5 of 64) received surgery. One of these patients received additional WBRT, one SFS, 3 did not receive additional RT. RT was applied as sole therapy in 68.8% (n = 44 of 64) patients at first recurrence. Of these patients 2 were treated by SFS, 27 by WBRT and 15 by radiosurgery.

At second recurrence, 88% of patients (n = 22 of 25) were treated by radiotherapy. 12% of patients did not receive any form of therapy at second recurrence. RT was applied as SFS in 4 patients, WBRT in 7 patients and radiosurgery in 11 patients.

A third intracerebral recurrence was observed in 12 patients. 6 of these patients received radiotherapy. One patient was treated by WBRT, the other five received radiosurgery.

Five patients experienced a fourth episode of intracranial recurrence. Two patients were treated by RT, receiving either WBRT or radiosurgery in reduced doses.

3 of the patients that developed LMD were not treated due to a reduced general health status. 4 patients received WBRT.

## Discussion

We analyzed the outcome of 119 patients with 123 resection cavities after surgical resection and local radiotherapy to the resection cavity in order to evaluate the efficacy of local radiotherapy in patients with an opening of the ventricles during surgery. No difference in local control, distant brain control, development of LMD or OS was noted when comparing patients with and without VO. The only difference observed between the two groups was a significantly increased rate of distant intraventricular metastases in patients with VO.

Radiosurgery and HFSRT are both practicable concepts to achieve local tumor control after surgical resection of brain metastases. A large randomized study demonstrated improved cognitive functioning for radiosurgery vs. whole brain radiotherapy yet at the price of reduced distant and local control [2]. HFSRT may overcome the disadvantage of the higher local failure rate as fractionation permits the use of increased safety margins to compensate for infiltrative growth and uncertainties in target volume due to volumetric changes of the cavity [22–24]. Multiple retrospective studies have demonstrated outstanding local control rates for resection cavities treated with HFSRT [6, 8, 10, 12]. The one year local control rate of a cohort from our clinic and a combined analysis with another institution applying a similar radiation scheme of 30/35 Gy in 5 Gy fractions was 88% and 80.5% [6, 8]. This is noticeably superior to the one year local control rates of 60.5% and 72% reported by Brown et al. and Mahajan et al. with radiosurgery and comparable to the WBRT control group [2, 3]. Other groups have shown similar local control rates with different fractionation schemes [26, 27]. Furthermore, the safety profile of HFSRT seems to be favorable compared to radiosurgery, particularly for large cavities [9, 28]. In comparison to WBRT, on the other hand, the risk of radionecrosis seems to be higher for HFSRT [2, 6, 11, 29, 30]. A potential downside to HFSRT and radiosurgery is the increased risk of leptomeningeal spread and lower distant brain control [2, 26, 30–32]. While localized distant progression can be easily salvaged after local radiotherapy to the resection cavity, LMD is a severe complication that considerably diminishes the patients’ survival [12, 33].

Surgical resection of brain metastases as well carries a risk of intracerebral tumor cell dissemination. LMD rates as high as 36% have been reported particularly in patients with piece meal resection of posterior fossa tumors [15–17]. An opening of the ventricles during surgery creates a connection between the resection cavity, the location with the highest risk for recurrence and the cerebrospinal fluid. A dissemination of tumor cells through the CSF could be a potential consequence. Therefore, the aim of this study was to assess whether local HFSRT of the resection cavity is a viable option in patients with resected brain metastases in which VO was necessary during surgery.

Neither distant brain control nor LMD development was significantly associated with an opening of the ventricles. Therefore, we assume local radiotherapy to be sufficient in these cases. However, even if numbers are not statistically different, it seems that the 2-year intracranial control in the VO subgroup is arithmetically lower. The total number of events is clearly limited, possibly disguising differences.

The development of distant intraventricular metastases was significantly increased in patients with an opening of the ventricles pointing towards a potential dissemination of individual tumor cells without the capacity to induce disseminated LMD. Since adequate therapeutic strategies for intraventricular metastases such as resection or local radiotherapy exist and neither OS nor distant control were affected by the development of distant intraventricular metastases, no change in target volume definition seems necessary due to our findings [34–36]. An alternative explanation for the higher distant intraventricular failure rate is the larger preoperative metastatic diameter of metastases with VO, as larger metastases are associated with increased distant failure rates [28]. Further thought should be given to target volume definition in this particular situation. Recently, contouring guidelines for stereotactic radiotherapy to resection cavities have been established recommending an additional margin of 10 mm for metastases with dural contact and up to 5 mm for metastases with venous sinus contact [37]. Whether an additional ependymal margin is of value cannot be answered by this study, since our standard contouring concepts add 4–5 mm margins as we apply fractionated radiotherapy. A potential alternative is preoperative radiotherapy which permits an easier definition of the target volume [38]. However, preoperative radiotherapy also is accompanied by potential pitfalls such as the absence of histopathological specimen at the time of radiotherapy and a possible increase in wound complications [41].

A comparable study has been conducted by Adeberg et al. for patients with glioblastoma that experienced an opening of the ventricles during surgical resection. Similarly to our results, no increased incidences of distant brain recurrence were noted in the study [39]. However, a number of studies demonstrated higher rates of LMD in high grade glioma patients after VO [20, 42, 43]. Moreover, the dissemination pathways of glioblastoma and metastases are likely different.

With roughly 6% the overall incidence of LMD was relatively low. LMD occurred more frequently in certain histologies such as NSCLC, melanoma and breast cancer, which is in line with findings by other groups [40]. The incidence of LMD was not significantly higher in patients with infratentorial metastases which might be due to improved surgical techniques.

Limitations to our study include the low incidence of VO and subsequent small patient and event numbers. Furthermore, we lack information on the systemic therapies applied. Therefore, we cannot affirm that the distribution of systemic therapies such as immunotherapies, that might influence the outcome, including the risk of intracranial dissemination, was equal between the groups.

The fact that none of the patients with VO developed LMD, nevertheless, underlines the hypothesis that local radiotherapy is viable in patients with VO during surgical resection of brain metastases. However, further studies should be conducted to clarify the subject.

## Conclusion

VO during neurosurgical resection did not affect the outcome after HFSRT of the resection cavity in patients with brain metastases. Particularly, the incidence of LMD was not increased in patients receiving local HFSRT after VO. HFSRT can therefore be offered independently of VO as a local treatment of tumor bed after resection of brain metastases.

## Data Availability

The datasets generated and/or analysed during the current study are not publicly available due to preservation of privacy but are available from the corresponding author on reasonable request.

## Abbreviations

BED: Biologically effective dose
CTV: Clinical target volume
CSF: Cerebrospinal fluid
HFSRT: Hypofractionated stereotactic radiotherapy
LMD: Leptomeningeal disease
LC: Local recurrence
OS: Overall survival
PTV: Planning target volume
VO: Ventricular opening
WBRT: Whole brain radiotherapy
